# Emergency medicine in the United Arab Emirates

**DOI:** 10.1186/1865-1380-7-4

**Published:** 2014-01-08

**Authors:** Saleh Fares, Furqan B Irfan, Robert F Corder, Μuneer Abdulla Al Marzouqi, Ahmad Hasan Al Zaabi, Marwa Mubarak Idrees, Michael Abbo

**Affiliations:** 1 Department of Emergency Medicine, Zayed Military Hospital, P.O. Box: 8313, Abu Dhabi, United Arab Emirates; 2 Department of Emergency Medicine, Harvard-Affiliated Disaster Medicine/Emergency Management Fellowship Program, One Deaconess Road, West Campus Clinical Center, 2nd Floor, Boston, MA 02215, USA; 3 Rashid Hospital Trauma Center, Dubai Health Authority (DHA), PO Box 4545, Dubai, United Arab Emirates; 4 Department of Emergency Medicine, Tawam Hospital, PO Box 15258, Al Ain, Abu Dhabi, United Arab Emirates; 5 Department of Emergency Medicine, University of Maryland School of Medicine, 6th Floor, Suite 200, 110 South Paca Street, Baltimore, MD 21201, USA; 6 College of Medicine and Health Sciences, United Arab Emirates University, P.O. Box 17666, Al Ain, United Arab Emirates; 7 Emergency Department, Rashid Hospital Trauma Center, PO Box 4545, Dubai, United Arab Emirates

## Abstract

It has been a decade since emergency medicine was recognized as a specialty in the United Arab Emirates (UAE). In this short time, emergency medicine has established itself and developed rapidly in the UAE. Large, well-equipped emergency departments (EDs) are usually located in government hospitals, some of which function as regional trauma centers. Most of the larger EDs are staffed with medically or surgically trained physicians, with board-certified emergency medicine physicians serving as consultants overseeing care.

Prehospital care and emergency medical services (EMS) operate under the auspices of the police department. Standardized protocols have been established for paramedic certification, triage, and destination decisions. The majority of ambulances offer basic life support (BLS/Type 2) with a growing minority offering advanced life support (ALS/Type 3).

Medicine residency programs were established 5 years ago and form the foundation for training emergency medicine specialists for UAE.

This article describes the full spectrum of emergency medicine in the UAE: prehospital care, EMS, hospital-based emergency care, training in emergency medicine, and disaster preparedness. We hope that our experience, our understanding of the challenges faced by the specialty, and the anticipated future directions will be of importance to others advancing emergency medicine in their region and across the globe.

## Background

**Figure 1:**
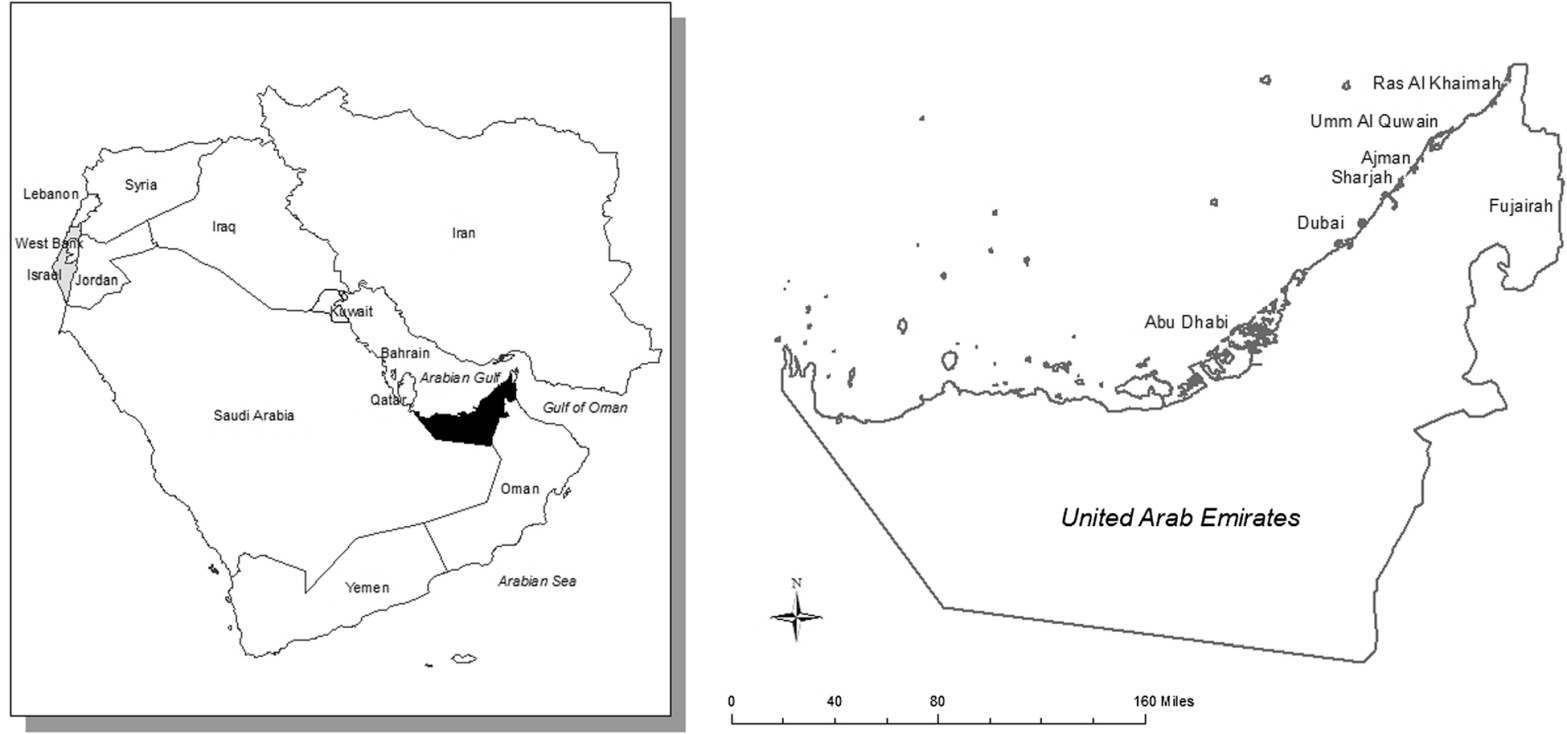
The location of the United Arab Emirates in the Middle East.

The United Arab Emirates (UAE), formed in 1970, is a constitutional federation of seven states called “emirates.” It is the size of the state of Maine and is situated in the southeastern tip of the Arabian Peninsula. On its borders are Oman and Saudi Arabia (Figure 1). The emirates are Abu Dhabi, Dubai, Sharjah, Ajman, Umm Al-Quwain, Ras Al-Khaimah, and Fujairah; Abu Dhabi is the capital and the largest emirate and Dubai is the center of the tourist industry. After the discovery of oil in the early 1960s, the UAE developed rapidly since the area holds just under 10% of the world’s oil. The country has diversified its economy and has become a major industrial, tourism, trade, and financial hub [1]. It has experienced high population growth, mainly due to the immigration of expatriate workers. According to the last census, the population of the UAE in 2010 was estimated at 8.19 million, which is expected to double by 2029. Interestingly, less than 20% of the population are UAE nationals; the majority are expatriates [2]. Two-thirds of the population lives in Abu Dhabi, Dubai, and Sharjah. The median age is 37, with less than 1% of the population over 65 years of age.

Health care in the UAE has undergone a similarly rapid development. Despite this rapid pace of development, or perhaps because of it, there is a paucity of published reports describing health care in the UAE. This report describes the specialty of emergency medicine, addressing its structure, workforce, training, and the challenges facing it in this rapidly developing, high-income country.

## Methods and discussion

Most of the information in this article was provided by physicians practicing emergency medicine in the UAE. Information about areas outside the emirates was obtained through informal interviews of physicians working in emergency departments (EDs) in those locations. Three of the authors are heads of departments and have extensive knowledge of emergency medicine in the largest emirates, Dubai and Abu Dhabi. They are also heavily involved with national activities through the Emergency Medicine Society.

### Health care in the UAE

The current high standard of health care evolved rapidly over a few decades. Key health indicators, such as life expectancy and infant mortality rates, which were below average, are currently are at par with those of developed countries [3]. These health statistics reflect a commitment by the government to increase public spending and investment in health care infrastructure and services.

To appreciate the rapidity of these changes, one may look at the health care infrastructure. In 1970, the country had 7 public hospitals; today, there are 40. Half of the 60 private hospitals were built during the past two decades. Hospital bed availability, at 1.9 per 1,000 population in 2008, is rapidly increasing with the opening of several new hospitals (see below). There are 243 government-run primary health care centers (PHCs) [4]. Despite the large number of PHCs, EDs in the UAE are faced with high numbers of minor complaints compared with other developed countries [5]. In recent data published by the Health Authority–Abu Dhabi (HAAD) in the local media, more than 75% of cases in EDs in Abu Dhabi are non-urgent. This situation was made more challenging by the shortage of physicians trained in emergency medicine: in 2008, there were only 1.93 physicians per 1,000 population in the UAE [6,7].

### Government oversight of health care

The Ministry of Health manages and operates health care facilities throughout the UAE and is responsible for setting up and implementing policies, regulations, and standards toward achieving comprehensively integrated health care systems [8]. However, health care is decentralized, in that each emirate has its own health authority to allow local decision making. In 2007, the General Authority for Health Services of Abu Dhabi was restructured into two entities: HAAD, Abu Dhabi’s regulative and health policy body, and Abu Dhabi Health Services Company, an independent, public joint stock company that owns and operates hospital facilities, ambulatory clinics, and primary health care clinics in Abu Dhabi [9,10]. Dubai’s Department of Health and Medical Services was also restructured into the Dubai Health Authority in 2007 to oversee an integrated health care system [11].

### Western hospital affiliation

Decentralization of the health care sector has positively affected the system. Doors were opened to western health care experts to advance care in each emirate. The investment and the efforts to achieve excellence in health care and research led to numerous affiliations and contracts awarded to international institutions [12]. Johns Hopkins Medicine International runs Al Rahba and Corniche Hospitals in Abu Dhabi as well as Tawam Hospital in Al Ain. The Cleveland Clinic manages Sheikh Khalifa Medical City in Abu Dhabi and is currently commissioning and staffing a tertiary referral hospital that will be operational in 2015. This will be a stand-alone, state-of-the-art facility staffed by physicians and nurses certified by American professional organizations. Ras Al-Khaimah, a northern emirate, recently awarded management of its hospital to the Methodist Healthcare Group.

In Dubai, Healthcare City was created with the backing of international affiliates, including the Dubai Harvard Foundation for Medical Research, Boston University Postgraduate Dental Training Program, and Moorfields Eye Hospital.

### Emergency medicine specialists

In 2000, hospital administrators in Abu Dhabi and Dubai began to recruit emergency medicine-trained physicians to their facilities. Prior to that time, emergency medical treatment was provided in accident and emergency (A&E) departments in public hospitals [13], which were staffed by general medical officers trained in medical or surgical disciplines. These physicians would refer cases to specialists for their opinion and treatment. Multisystem complaints would generate a number of referrals. Responsibility for care often fell between these specialists, causing long length of stays in the A&E departments. The size of these emergency centers ranged from primary health care facilities with limited hours of service to hospital-affiliated wards with 24-hour access to radiology and laboratory support.

This structure changed in 2000 with the establishment of an ED in Abu Dhabi’s Sheikh Khalifa Medical Center (the UAE’s largest hospital at the time), where a group of western emergency physicians oversaw care. A few years later, in 2006, Dubai’s Rashid Hospital Trauma and Emergency Center opened its doors and followed suit. Several EDs in the larger hospitals across the country have adopted similar models. The transition to EDs brought the first generation of western board-certified emergency medicine physicians to the area. These pioneers took the first steps toward establishing emergency medicine in their respective hospitals. The challenges were not dissimilar to those seen across the United States in the early 1970s. Among their achievements was the demonstration that emergency medicine specialists can improve the delivery of emergency care, as has been demonstrated in many other countries [14]. Those first emergency medicine physicians working in the UAE recognized that sustainability depended on starting local emergency medicine training programs and forming an accreditation body that could award board certification in EM. The UAE’s first emergency medicine conferences and activities followed [15,16].

In August 2012, a group of emergency physicians established the Emirates Society of Emergency Medicine (ESEM). Its vision is to develop and maintain high standards of emergency medicine practice; to function as the networking body that connects emergency medicine providers across the UAE; to promote collaboration among ED doctors, nurses, and paramedics through scientific conferences, workshops, and meetings; and to encourage emergency medical research. ESEM promotes emergency medicine to medical students and interns and has established subcommittees that represent subgroups for residents, nurses, and paramedics within the emergency medicine specialty. ESEM also raises public awareness of the specialty of emergency medicine throughout the region as well as of ED utilization, injury prevention, and public safety. ESEM held its first annual conference in Dubai in May 2013 and continues to grow rapidly in several aspects, including emergency medicine education. Emergency medicine’s progress in the UAE has been recognized and commended by international entities. Acceptance by local and international emergency medicine entities has set the stage for further growth, positioning the UAE to become a leader in emergency medicine training and practice.

### Emergency medical services

Extension of emergency care into the prehospital setting provides opportunities for earlier initiation of evidence-based therapies, rapid access to aggressive treatment strategies, and coordination with capable centers for efficient delivery of care. This approach is supported by numerous international guidelines, which emphasize the need for early activation of emergency medical services (EMS) for patients with time-sensitive complaints (e.g., acute coronary syndrome) [17-19].

Prehospital care has been one of the most challenging aspects of the emergency spectrum in the UAE. EMS in the UAE are largely organized and run by the police departments of the respective emirates [12,20-22]. Each emirate has a single emergency response activation telephone number—“999” or “998” [12,23,24]. Calls are received and ambulances are dispatched by a police dispatch center, which is supervised by police physicians and paramedics not trained in emergency medicine [22,25]. The Ras Al-Khaimah Police Department recently installed a new system in its operations center to provide increased capacity and efficiency and faster ambulance response times [26]. In the larger cities, transport decisions are based on triage protocols; in smaller towns and rural areas, patients are usually transported to the nearest medical facility [12].

Larger public and private medical facilities also own and run ambulance services [25]. Their ambulances are used primarily for interhospital transfers, while police-run EMS ambulance crews provide acute prehospital care. At the present time, there is minimal coordination between these groups and many gaps exist in the services they provide.

### EMS transition

During the past few years, several mass casualty incidents served to identify deficiencies within the EMS system and attracted significant attention in overcoming them. In the Emirate of Abu Dhabi, numerous collaborative efforts were established between the HAAD and the police department to coordinate efforts and upgrade the EMS system. One aspect of this initiative was the adoption of guidelines from the Joint Royal Colleges Ambulance Liaison Committee, which are to be implemented throughout Abu Dhabi [27]. Another important step was the establishment of the National Ambulance Company in 2010 [28]; based in Abu Dhabi, it aims to be the leading national provider of emergency prehospital care in the UAE [28]. In 2006, the Dubai Police Department and the Department of Health and Medical Services combined their services to create the Center of Ambulance Services [12]. Similar efforts are underway in other emirates to involve health departments and police departments in the management and operations of EMS resources. Ambulances operated by the police departments are based at “rescue and emergency centers” located in populated areas. The Sharjah Police Department, for example, has established 18 rescue centers throughout the emirate, including two sea-based centers [20].

### Paramedic training

Standardization of paramedic levels and competencies is lacking in most of the smaller emirates. In a non-published report obtained with permission from the National Ambulance Company about the status of EMS in the northern emirates (Sharjah, Ajman, Umm Al Quwain, Ras Al Khaimah, and Fujairah), Sharjah was in a better state operationally than the other emirates, having 103 prehospital care providers compared with 17 in Ajman, 21 in Ras Al Khaimah, 13 in Fujairah, and none in Umm Al Quwain. Qualifications for paramedics range from a bachelor’s degree or diploma in nursing to certification in specific courses such as prehospital trauma life support, advanced cardiac life support, and pediatric advanced life support. In contrast, larger emirates, specifically Abu Dhabi and Dubai, have taken bigger steps toward defining prehospital care as a profession. They have established a standardized curriculum that takes into consideration the individual’s level of education, training, and experience. A paramedic is classified as an emergency medical technician (EMT), EMT-Basic, EMT-Intermediate, and EMT-Paramedic based on his qualifications. This classification is currently under revision, as stated earlier. The licensing and certification of prehospital personnel are provided by health authorities of the respective emirates, i.e., HAAD and the Dubai Healthcare Authority [12,25]. HAAD classifies ambulances into those providing basic life support (BLS/Type 2) or advanced life support (ALS/Type 3), the latter carrying cardiac monitors and controlled drugs.

### EMS equipment

In Abu Dhabi, approximately 80% of the ambulances are fully equipped ALS/Type 3 vehicles [25]. Other land-based EMS vehicles include fast-responder units, motorcycles, mobile intensive care units, and bus-based mobile hospitals for mass casualty incidents [12,25]. Air EMS are operated by a different department called the Air Wing, creating a gap in coordination with land services. These resources include fixed-wing and rotor services (e.g., Bell 412 and Augusta Westland 139 helicopters) [22,25]. The aircraft are staffed by crews consisting of pilots, first aiders, paramedics, and occasionally doctors [25].

Recent studies revealed a lack of awareness in the general population about how to activate and utilize EMS resources. Fares and colleagues [29] reported that the use of EMS by only 17% of patients with acute coronary syndromes was shown to be lowest in the region compared with 23% in Bahrain, 30% in Qatar, and 37% in Oman.

The quality of prehospital care varies considerably among the emirates for several reasons, the most significant of which are differences in EMS authorities, resulting in a lack of coordination and synergy. Another factor is the limited availability of EMS-trained professionals in the country.

### Hospital-based emergency care

Medical care for minor illnesses and injuries is provided at PHCs staffed by family or internal/general medicine physician centers. Most PHCs are walk-in facilities, similar to urgent care centers in the United States. The larger centers offer basic radiologic, diagnostic, and laboratory services; smaller PHCs depend on offsite laboratory and diagnostic services. The PHCs maintain good communication with public hospitals and integrate care with them. Patients requiring care beyond what can be provided at a PHC are transferred to EDs in secondary and tertiary hospitals. There are no financial disincentives to accepting transfers from PHCs, as may be the case in other countries.

The large public hospitals in the UAE provide services free of charge for all conditions except minor emergencies. However, this largesse could change in the future if national insurance becomes mandated for all. Private hospitals require upfront payment.

Statistics on ED utilization in the UAE are not available but are being collected by government entities. There are a reported 124 hospitals in the 7 emirates: 40 in Abu Dhabi, 40 in Dubai, 12 in Sharjah, 12 in Ajman, 11 in Ras Al Kaimah, 6 in Fujairah, and 3 in Umm Al Quwain [30].

Large EDs (seeing more than 30,000 patients per year) physically resemble those in the United States. Some have designated areas for pediatric medicine, obstetric emergencies, and observational medicine [16] and have adopted international models for triage, patient monitoring, transfer, trauma, and critical care. Most medical and surgical specialties are available. Where subspecialty care is not available, hospitals have agreements with government hospitals to provide the service.

### Trauma care

Trauma patients in the UAE are younger than those in North America, reflecting differences in demographics. The median age of trauma patients in the UAE is 30 compared with 37 in North America. Most trauma patients in the UAE have sustained blunt mechanisms of injury; penetrating trauma is rare. In 2012, the Dubai Trauma Center treated four victims with gunshot wounds (Dr. Michael Abbo, personal observation). According to the World Health Organization, residents of the UAE are seven times more likely to die in a car crash than are citizens of the United Kingdom (32 deaths vs. 4 per 100,000 inhabitants). Since the 1980s, motor vehicle crashes have been the second most common cause of death in the UAE after cardiovascular diseases, with their totals increasing steadily. The passage of strict highway safety laws, the imposition of significant fines for speeding, and several government initiatives are expected to limit this increase [3].

Sheikh Khalifa Medical City and Mafraq Hospital in Abu Dhabi and Rashid Hospital Trauma Center in Dubai are the designated regional trauma hospitals. Using data from a German trauma registry, we calculated that the Dubai Trauma Center has mortality and morbidity rates that are comparable to those of European trauma centers (Dr Michael Abbo’s personal communication). A trauma system for the Emirate of Abu Dhabi is being developed, with plans to extend it to the whole of the UAE over the next decade [31]. In this project, trauma care is seen as being delivered on a “continuum” that begins with prevention but also extends from the time of injury through to rehabilitation. The initiative is based on the components suggested by the Committee on Trauma of the American College of Surgeons [32]. Several successes can already be noted: the introduction of injury prevention programs and the launch of a trauma registry.

### Staffing

EDs in the UAE are staffed mostly with expatriate physicians trained in medicine or surgery. The larger EDs also have residents, house officers/interns, and medical students. It is estimated that there are 1,000 physicians working in EDs in the UAE. About 50 of them are emergency medicine physicians with western qualifications or board certification; as can be expected, these specialists are based in the larger, well-established EDs and trauma centers in the UAE [12]. ED nurses and technicians are also mostly expatriates, the majority being from the Philippines or India. English is spoken in the workplace. The ancillary staff and nurses have a variety of nursing qualifications and ED experience, depending on their home countries. The Ministry of Health and respective health authorities are responsible for assessing their competency and licensing. There are no common mandates in the emirates for patterns or ratios of physician and nurse staffing.

### Graduate medical education

Medical school education in the UAE follows the European model [12]. After completion of the US equivalent of high school, students attend a 5-year medical school, followed by a mandatory “internship” or transitional year. Specialty training in emergency medicine (residency) begins on completion of the internship year.

The UAE has five medical schools, and most of them receive government funding. They are co-educational, except for Dubai Medical College for Girls, a private institution. Until recently, the Faculty of Medicine and Health Sciences Medical School (United Arab Emirates University), located in Al Ain, admitted only Emirati students, but it is now open to non-Emiratis too; it is government funded and tuition is free. The other medical schools are Gulf Medical University in Ajman; Ras Al-Khaimah Medical and Health Sciences University, which was established by the Ras Al Khaimah Human Development Foundation in 2006 [33]; and the College of Medicine at University of Sharjah. Together, these schools graduate approximately 400 medical students each year.

### Emergency medicine residency training

UAE offers unique opportunities for emergency medicine training. Hospitals are well funded and equipped with the most modern diagnostic and therapeutic tools. Trainees are exposed to a large range of pathology ordinarily not encountered in any one country. The diversity of transient nationalities living in UAE exposes trainees to diseases endemic to their countries of origin. The presence of one of the world’s busiest airports provides excellent exposure to travel-related emergencies. The experience that can be gained in the management of blunt trauma is also unmatched; the large numbers of victims of blunt trauma come from construction site incidents and motor vehicle crashes.

The country now has three hospital-based emergency medicine residency programs, which are independent of each other, each having its own curriculum. The first was started in 2007 at the Rashid Hospital Trauma Center, a modern, public, state-of-the-art hospital with 140,000 patient visits per year. This 5-year program (the fifth devoted to research or an elective) has 38 residents. The other two programs are at Tawam Hospital and Sheikh Khalifa Medical City in Abu Dhabi. The 4-year program at Tawam Hospital program has 24 residents and is affiliated with the UAE University. The emergency medicine residency at Sheikh Khalifa Medical City has 9 residents. A fourth emergency medicine training program is anticipated to start at Zayed Military Hospital in Abu Dhabi in 2014.

Residency training in the UAE follows the North American model, with structured academic days, morbidity and mortality conferences, journal club, and grand rounds. Residents are expected to complete all certificate courses such as advanced cardiac life support, pediatric advanced life support, and advanced trauma life support by the end of their training. They are required to sit for a year-end in-service examination. Medical school graduates apply for residency through the National Resident Matching Program. Statistics collated from residency directors show a one-in-four acceptance rate. Preference is given to Emirati students, who made up 40% of last year’s incoming group of residents. Other nationalities include Indians, Pakistani, and Middle East as well as North African residents. Women are the majority in each class. All programs are currently accredited by the Arab Board of Emergency Medicine, which is modeled after the American Board of Emergency Medicine. The exit exam has an oral and a written component. The benefits of the Arab Board are currently being debated; the board is yet to be recognized internationally, against other Board certifications, and there is a lack of standardization among the different programs in hosting countries. Other boards that are being considered as the certifying organization for emergency medicine physicians in the UAE are the Saudi Board, ACGME International, or a new certification board for the country; each has its proponents and opponents. As an example, the Abu Dhabi programs have adopted the requirements of the Accreditation Council for Graduate Medical Education (ACGME), which will allow local trainees to sit for the American Board of Emergency Medicine examination. The Tawam program is on its way to receiving accreditation by ACGME International.

By the end of 2015, these programs will graduate 20 emergency medicine residents each year. Consolidation of all programs under one umbrella is being considered, and this would facilitate the development and acceptance of a national curriculum and possibly the creation of an academic department of emergency medicine at the UAE University.

### Disaster preparedness

Over the past decade, disasters, both manmade and natural, have made disaster medicine and emergency management priorities for hospitals and health care systems [34,35]. With the UAE being a major oil-rich financial and tourism hub, preparedness for incidents such as oil spills, plane crashes, and even terrorist attacks forms an important component of the country’s comprehensive emergency medical system. A number of major motor vehicle crashes created mass casualty incidents during the past few years, capturing the attention of the medical community as well as the community at large. In 2008, on what has become known as Fog Tuesday, a 200-vehicle pile-up in heavy fog during the morning rush-hour on the Abu Dhabi-Dubai highway killed 4 people, injured 350, and left 20 cars on fire [36]. Natural disasters also threaten the UAE: during the past 2 decades, the emirates have witnessed earthquakes, floods, and tropical storms [37]. The UAE has 734 km of coastline, making the majority of its population and cities vulnerable to tsunamis [38].

Authorities in the UAE are focusing on all phases of the emergency management continuum, after studying the large number of emergencies and disaster victim contingencies [36] and their overwhelming financial burden. The establishment of the National Crisis and Emergency Disaster Management Authority (NCEMA) in 2007 represented a paradigm shift from a “relief-centric” post-event management policy into a proactive, multidisciplinary, and holistic approach, covering all phases of mitigation, preparedness, response, and recovery. The NCEMA, a national body under the auspices of the Higher National Security Council of the UAE, is responsible for coordinating and ensuring the preparedness of all organizations involved in the management of emergencies and crises in the UAE [39].

The UAE has taken the lead in the region on disaster and emergency management. In 2012, NCEMA hosted the Third Crisis and Emergency Management Conference, which was held in Abu Dhabi [39]. Emergency management teams coordinated by NCEMA have been set up in all emirates to raise the level of preparedness and emergency response [39,40]. One of the world’s largest disaster management facilities, Tawazun Disaster Management City, is being built in Abu Dhabi. It will be a unique facility, offering multi-agency training for emergency, crisis, and disaster management for all types of natural and man-made disasters [41]. A study using geomatics to assess tsunami risk along the eastern coast of the UAE was recently carried out in Fujairah [42]. Umm Al Quwain has developed a plan for advanced emergency response to hazards, based on a study by the Roadway, Transportation and Traffic Safety Research Centre at UAE University in collaboration with the Emergency Centre of Lancashire, United Kingdom [42,43]. In 2011, Dubai was one of three Arab cities selected for testing of the disaster reduction and preparedness criteria under a global platform provided by the United Nations International Strategy for Disaster Reduction [44]. The country’s “paradigm shift” is also reflected in individual ED disaster preparedness plans and yearly disaster drills.

### Challenges

The challenges faced in emergency medicine in the UAE are similar to those faced in the United States 30 years ago. Emergency department positions in the UAE were jobs of last resort for physicians who were not successfully integrated into other specialties. The salaries were less than those for other specialties; recognition of the field was minimal, as were opportunities for career advancement in emergency medicine.

The UAE still has a severe shortage of emergency medicine-trained practitioners. Currently, less than 10% of practicing emergency physicians in the UAE are residency trained. Most physicians working in hospital EDs have training in medicine or surgery, so they are accustomed to referring complex patients to appropriate services. Tasking a handful of emergency medicine consultants to change entrenched mindsets to enable untrained emergency medicine physicians to diagnose, stabilize, and treat complicated patient presentations will be a significant, possibly insurmountable, challenge. It is more likely that system changes that recognize and promote recent emergency medicine residency graduates will accomplish these goals.

The shortage of emergency medicine-trained, board-certified consultants in the UAE has a negative impact on the quality of emergency medicine training. The consultants who are available in the UAE have little to no available time for teaching and research. This imbalance could be aggravated in the future if residents certified by the Arab Board are not recognized at the level of emergency medicine-certified physicians in North America. Arab physicians might decide to leave the UAE to enter residencies in North America, with the hope of being instated at a consultant/attending level upon their return. Some could decide to take jobs outside the country after completing their training. The lack of a defined pathway to consultant/attending status in the UAE and the perception that training is better in the west is likely to contribute to this attrition.

### The future

The need to sustain ED capabilities and fulfill the mission of emergency medicine in the face of a growing population, declining resources, and financial restraints pose significant challenges in the UAE—challenges that emergency medicine providers are uniquely able to manage. In addition to ensuring patient safety and good clinical outcomes for acutely ill patients, emergency medicine-trained physicians will have a positive influence on the health status of our communities, resource utilization, and the development of prehospital systems, as well as hospital and public health policies. Adopting best practices and enhancing the skills of emergency care providers will certainly help strengthen the specialty’s reputation.

## Conclusions

In the UAE, emergency medicine has come a long way in a short time. The specialty is advanced in its training programs and systems of care compared with most Middle East countries, but it is still in its infancy compared with North American systems. Considering the resources available in the UAE and the variety and severity of disease and injury presentations, it is difficult to envision a more fertile ground for emergency medicine training and practice. The creation of the Emirates Society of Emergency Medicine, the availability of residency training sites, and the development of fellowship programs, will advance the specialty even further.

## Abbreviations

EDs: Emergency departments; EMS: Emergency medical services; EMT: Emergency medical technician; HAAD: Health Authority–Abu Dhabi; NCEMA: National crisis and emergency disaster management authority; PHCs: Primary health care centers; UAE: United Arab Emirates.

## Competing interests

The authors declare that they have no competing interests.

## Authors’ contributions

Authors SF, FI, RC and MA equally contributed to the work by gathering data, literature review, manuscript drafting and reviewing. Authors MAA, AHA amd MMI carried out field visits and data gathering from selected government entities. All authors read and approved the final manuscript.

## References

[B1] United Arab Emirates 2013 [http://en.wikipedia.org/wiki/United_Arab_Emirates#cite_note-36]

[B2] The National Population leaps to 8.19 million 2010 [http://www.thenational.ae/news/uae-news/population-leaps-to-8-19-million]

[B3] Regional Health Systems Observatory United Arab Emirates: Health System Profile 2006 EMRO, WHO

[B4] Library of Congress; Federal Research Division United Arab Emirates (UAE): Country Profile 2006 Washington DC: Library of Congress

[B5] Durand A-C Gentile S Devictor B Palazzolo S Vignally P Gerbeaux P Sambuc R ED patients: how nonurgent are they? Systematic review of the emergency medicine literature Am J Emerg Med 2011 7 333 345 10.1016/j.ajem.2010.01.003 20825838

[B6] Central Intelligence Agency The World Factbook, Middle East, United Arab Emirates 2012 [https://www.cia.gov/library/publications/the-world-factbook/geos/ae.html]

[B7] The Prospect Group Healthcare in the United Arab Emirates (UAE) 2012 [http://www.theprospectgroup.com/healthcare-in-the-united-arab-emirates-uae-81878/]

[B8] Ministry of Health, UAE Hospitals and Medical Centers 2012 http://www.moh.gov.ae/en/About/Pages/ServiceProviders.aspx

[B9] Abu Dhabi Health Services Co. P.J.S.C, SEHA Formation 2008 [http://www.seha.ae/seha/en/Pages/SehaFormation.aspx]

[B10] Sheikh Khalifa Medical City, A SEHA Health System Facility Healthcare in Abu Dhabi 2012 [http://www.skmc.ae/en-us/About/Pages/HealthcareinAbuDhabi.aspx]

[B11] Government of Dubai, Dubai Healthcare Authority Who We Are 2012 [http://www.dha.gov.ae/en/Aboutus/Pages/WhoWeAre.aspx]

[B12] Partridge R Abbo M Virk A Emergency medicine in Dubai, UAE Int J Emerg Med 2009 7 3 135 139 10.1007/s12245-009-0122-y 20157462 PMC2760702

[B13] Dubai Health Authority Our History 2012 [http://www.dohms.gov.ae/En/aboutus/pages/ourhistory.aspx]

[B14] Birkhahn RH Gaeta TJ Tloczkowski J Mundy T Sharma M Bove J Briggs WM Emergency medicine-trained physicians are proficient in the insertion of transvenous pacemakers Ann Emerg Med 2004 7 4 469 474 10.1016/j.annemergmed.2003.09.019 15039689

[B15] Dubai Health Authority (DHA) Dubai Residency Training Programme 2013 [http://www.dha.gov.ae/EN/SectorsDirectorates/Directorates/MedicalEducation/Services/ResidencyProgram/Documents/DRTP%20Uuer%20Manual.pdf]

[B16] Sheikh Khalifa Medical City, A SEHA Health System Facility Residency Programs [http://www.skmc.ae/en-us/Careers/Pages/ResidencyPrograms.aspx]

[B17] Antman EM Hand M Armstrong PW Bates ER Green LA Halasyamani LK Hochman JS Krumholz HM Lamas GA Mullany CJ Pearle DL Sloan MA Smith SC Jr Anbe DT Kushner FG Ornato JP Jacobs AK Adams CD Anderson JL Buller CE Creager MA Ettinger SM Halperin JL Hunt SA Lytle BW Nishimura R Page RL Riegel B Tarkington LG Yancy CW 2004 Writing Committee Members 2007 Focused Update of the ACC/AHA 2004 Guidelines for the Management of Patients With ST-Elevation Myocardial Infarction: a report of the American College of Cardiology/American Heart Association Task Force on Practice Guidelines: developed in collaboration With the Canadian Cardiovascular Society endorsed by the American Academy of Family Physicians: 2007 Writing Group to Review New Evidence and Update the ACC/AHA 2004 Guidelines for the Management of Patients With ST-Elevation Myocardial Infarction, Writing on Behalf of the 2004 Writing Committee Circulation 2008 7 2 296 329 10.1161/CIRCULATIONAHA.107.188209 18071078

[B18] Pollack CV Jr Braunwald E 2007 update to the ACC/AHA guidelines for the management of patients with unstable angina and non-ST-segment elevation myocardial infarction: implications for emergency department practice Ann Emerg Med 2008 7 5 591 606 10.1016/j.annemergmed.2007.09.004 18037193

[B19] Bassand J-P Hamm CW Ardissino D Boersma E Budaj A Fernández-Avilés F Fox KA Hasdai D Ohman EM Wallentin L Wijns W Task Force for Diagnosis and Treatment of Non-ST-Segment Elevation Acute Coronary Syndromes of European Society of Cardiology Guidelines for the diagnosis and treatment of non-ST-segment elevation acute coronary syndromes Eur Heart J 2007 7 13 1598 1660 17569677 10.1093/eurheartj/ehm161

[B20] Khaleej Times Eight More Rescue Units to be Built in Sharjah 2011 [http://www.thefreelibrary.com/Eight+more+rescue+units+to+be+built+in+Sharjah.-a0250792095]

[B21] Khaleej Times Rescue and Ambulance Centre Opened in Ajman 2011 [http://www.khaleejtimes.com/darticlen.asp?xfile=data/theuae/2011/August/theuae_August232.xml&section=theuae]

[B22] Smith RR Trunkey D International Trauma Site Visit Repor 2009 Abu Dhabi, UAE: American College of Surgeons

[B23] Essential Telephone Numbers in Ajman 2009 [http://gulfnews.com/uaessentials/residents-guide/information/essential-telephone-numbers-in-ajman-1.442148]

[B24] Abu Dhabi eGovernment Gateway Citizen - Emergency Numbers 2011 [https://www.abudhabi.ae/egovPoolPortal_WAR/appmanager/ADeGP/Citizen?_nfpb=true&_pageLabel=p6600&lang=en]

[B25] George A EMS Status Report Abu Dhabi 2009 Abu Dhabi, UAE : Health Authority – Abu Dhabi (unpublished report)

[B26] Khaleej Times New System Helps RAK Police take more Emergency Calls 2011 [http://www.khaleejtimes.com/kt-article-display-1.asp?section=theuae&xfile=data/theuae/2011/january/theuae_january309.xml]

[B27] JRCALC Welcome to the JRCALC website 2009 [http://jrcalc.org.uk/]

[B28] Abu Dhabi National Ambulance Company. National Ambulance 2012 [http://www.nationalambulance.ae/]

[B29] Fares S Zubaid M Al-Mahmeed W Ciottone G Sayah A Al Suwaidi J Amin H Al-Atawna F Ridha M Sulaiman K Alsheikh-Ali AA Utilization of emergency medical services by patients with acute coronary syndromes in the Arab Gulf States J Emerg Med 2011 7 3 310 316 10.1016/j.jemermed.2010.05.002 20580517

[B30] List of Hospitals in the United Arab Emirates [http://en.wikipedia.org/wiki/List_of_hospitals_in_United_Arab_Emirates]

[B31] Griffiths JL Estipona A Waterson JA A framework for physician activity during disasters and surge events Am J Disaster Med 2011 7 1 39 46 21466028

[B32] American College of Surgeons Resources for the Optimal Care of the Injured Patient 2006 Chicago, Illinois: ACS

[B33] RAK Medical & Health Sciences University 2011 [http://www.rakmhsu.com/]

[B34] Brevard SB Weintraub SL Aiken JB Halton EB Duchesne JC McSwain NE Jr Hunt JP Marr AB Analysis of disaster response plans and the aftermath of Hurricane Katrina: lessons learned from a level I trauma center J Trauma 2008 7 5 1126 1132 10.1097/TA.0b013e318188d6e5 19001986

[B35] De Ville de Goyet C Health lessons learned from the recent earthquakes and Tsunami in Asia Prehospital Disaster Med 2007 7 1 15 21 17484358 10.1017/s1049023x00004283

[B36] The National Fatal Accident Echoes Deadly Fog Pile-up 2010 [http://www.thenational.ae/news/uae-news/transport/fatal-accident-echoes-deadly-fog-pile-up]

[B37] Dhanhani HAG Duncan A Chester D United Arab Emirates: Disaster Management with Regard to Rapid Onset Natural Disasters 2010 Advanced ICTs for Disaster Management and Threat Detection: IGI Global

[B38] Jordan BR Baker H Howari F Tsunami Hazards Along the Coasts of the United Arab Emirates 2005 United Arab Emirates: Arabiancoast 2005 Papers: Dubai Municipality

[B39] The Supreme Council for National Security National Emergency Crisis and Disasters Management Authority NCEMA 2010 [http://www.ncema.gov.ae/Portal/en/about-ncema.aspx]

[B40] Fujairah Observer Fujairah Ruler Forms Emergency Response Team 2011 [http://www.fujairahobserver.ae/news/-fujairah-ruler-forms-emergency-response-team--4405.html]

[B41] Disaster Management Facility Underway 2011 [http://abu-dhabi-metro.com/featured/disaster-management-facility-underway]

[B42] Abdouli KA Tsunami Risk Assessment using Geomatics in Fujairah City, United Arab Emirates 2011 ProQuest, UMI Dissertation Publishing

[B43] Roadway Transportation & Traffic Safety Research Center 2004 United Arab Emirates: U.A.E. University [http://www.transport.ae/RTTSRC/archive.php]

[B44] Khaleej Times Dubai Joins Campaign for Disaster Reduction 2011 [http://www.khaleejtimes.com/Displayarticle09.asp?section=todaysfeatures&xfile=data/todaysfeatures/2011/October/todaysfeatures_October35.xml]

